# The Role of Mass Spectrometry in the Cannabis Industry

**DOI:** 10.1007/s13361-019-02164-z

**Published:** 2019-04-16

**Authors:** Ben Nie, Jack Henion, Imelda Ryona

**Affiliations:** 1 Advion, Inc., 61 Brown Rd., Ithaca, NY 14850 USA; 2 Q2 Solutions, LLC, 19 Brown Rd., Ithaca, NY 14850 USA

**Keywords:** Mass Spectrometry, cannabis, marijuana, hemp, LC/MS, LC/MS/MS, GC/MS

## Abstract

The focus of this critical insight article is a brief overview of analytical challenges the cannabis industry faces and how analytical chemists have new opportunities to demonstrate the merits of employing mass spectrometry for the chemical analysis of cannabis and its products. The current range of cannabis products extends from recreational use to medicines, edibles, beverages, and beyond. The standards employed to assure product quality, integrity, and safety are lacking compared to those currently used by the pharmaceutical, food, and beverage industries. This manuscript overviews some of the important analytical issues that exist for the growth and harvest of the cannabis plant to the production of a wide variety of its products. Currently, the topics of interest for safety in cannabis testing where mass spectrometry can play an important role include what are currently referred to as potency, pesticides, terpenes, heavy metals, and mycotoxins from bacteria. Since each state in the USA as well as several countries has their own regulations, the analytical opportunities and challenges vary depending upon which jurisdiction a laboratory is supporting. This Critical Insight report will suggest where mass spectrometry can play an important role and provide valuable input on these topics.

**Figure:**
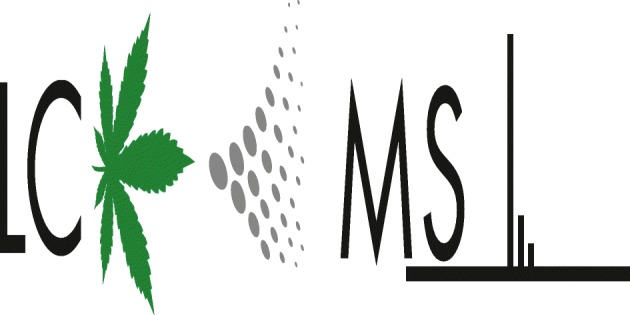
Graphical Abstract

Graphical Abstract

## Introduction

Although the cannabis industry is in its infancy, it is forecast to be a $22 billion industry by 2022 [1]. As a result, there is increasing commercial interest ranging from growers and product producers to investors in what is believed by some to be a very lucrative new market. Whenever such financal incentives exist, there will be those who endeavor to capitalize on them sometimes without regard to safety or integrity of the processes or products. Regulations are likely to evolve for standards of safety and integrity for cannabis products which are common to the pharmaceutical, food, beverage, and tobacco industries. In each of these latter mature industries, mass spectrometry is a routine, predominant player in developing products as well as assuring product quality and safety for the customer. As one who lived through the 1980s (JH) when the pharmaceutical industry began to explore and accept the analytical benefits of LC/MS and LC/MS/MS for drug metabolism and pharmacokinetic studies, it feels like a similar situation with the cannabis industry, e.g., dé·jà vu! Perhaps one of the reasons for the delayed experience within this industry is that marijuana has been classified as a Schedule I substance in the USA under the Controlled Substances Act since 1970.

Cannabis is the Latin word for marijuana and the stigma associated with the controlled substance notation has thwarted research on the potential medicinal value while producing the opportunity for abuse by those interested in its psychoactive properties. The term marijuana is a common name for the cannabis plant, *Cannabis sativa* L. which is a widespread species in nature. Although the word marijuana is often used, it is important to understand its meaning generally refers to the portion of the cannabis plant that contains higher levels of delta-9-tetrahydrocannabinol (THC). The term cannabis is the correct word for describing the plant and its products. The term hemp has historically referred to the use of cannabis as a fiber source which is very low in the psychoactive compound, delta-9-THC (< 0.3%), and has been used for many different products for thousands of years. The pharmacological effects of cannabis have been exploited for over 4800 years for recreational, medicinal, or religious purposes [2].

It has been less than 100 years since the chemicals in cannabis responsible for the production of some of its effects and the pharmacological actions were identified [3, 4]. Particularly noteworthy advances have been the discovery that cannabis is the source of a family of over 100 compounds known as phytocannabinoids, and that one of these compounds is delta-9-THC. This is the main psychoactive constituent of cannabis which for some people give the term “cannabis” a bad name. In spite of a long history of human and even animal use of cannabis, its use is illegal to this day in many states and other countries. Its effectiveness for treating various medical conditions has been reported in certain publications, but systematic, carefully conducted clinical trials are limited due to the stigma associated with the recent history of cannabis in today’s world. There are increasing numbers of testimonials to the effectiveness of “medicinal marijuana” to treat rare seizure disorders in young children and relieve symptoms of cancer therapy drugs, as well as relieving symptoms of anorexia and immune deficiency syndrome (HIV/AIDS) [5]. Of course cannabis has a popular following by those interested in the psychoactive effects of delta-9-THC which reportedly makes one feel “relaxed, happy, and even giddy.” In contrast to the considerable negative stigma associated with cannabis, the FDA recently approved the drug, Epidiolex, which is obtained by an efficient extraction of cannabidiol (CBD) from *Cannabis sativa* [6]*.* Some believe this recent development will be a huge boost for the acceptance of the non-psychoactive nature and benefits of CBD [7].

CBD has attracted interest from those experiencing insomnia, anxiety, and pain in addition to a growing list of other ailments [8]. Even beauty products have been reported such as mascara, lip balm, and eye cream that contain certain non-psychoactive cannabis components. One of the seemingly most dramatic recent reports is that the Coca-Cola Company is reportedly in discussions with the Canadian cannabis industry to infuse CBD into future Coca-Cola products [9]. This has been preceded by *Cannavines*, a California wine company which now sells a non-alcoholic red wine which has been infused with CBD [10]. Not to be outdone, *Sovereign Vines* in New York state has produced a novel wine which has been infused with certain “special tastes and flavors” from hemp [11]. From these reports, we can likely expect a wide diversity of new products containing cannabis and hemp [12] constituents going forward.

For those who understand the important role mass spectrometry can play as a sensitive and selective analytical technique, it is tempting to expect that it would be the technique of choice in this new industry which involves the chemical analysis of a natural product which contains hundreds of chemical entities across a very wide range of concentrations. Although LC/MS and GC/MS techniques are used in some cannabis applications (vide infra), other less definitive techniques such as LC/PDA (photodiode array) and GC/FID (flame ionization detector) are currently the popular analytical approaches [13]. The focus of this article is to contrast and compare the relative merits of these techniques applied to the needs of the cannabis industry and society as a whole. The sample types range from botanical plant materials and their oil products to edible foods including wines and beverages as mentioned above. The chemical constituents range from endogenous organic compounds including cannabinoids, terpenes, and flavonoids to exogenous compounds which include mycotoxins from mold growth to a wide range of pesticides and herbicides sometimes used in the cultivation of the plants. In addition, the determination of a substantial range of heavy metals must be measured as well as an impressive array of bacteria, molds, and fungi. To serve the growing need for chemical analysis of the many anticipated cannabis products, a number of contract service laboratories are springing up across the USA and elsewhere. Since each state has its own regulations on this industry, there is considerable inconsistency inter-state on how things are to be done. The authors’ purpose in this Critical Insight document is to present recommendations as to why mass spectrometric techniques should be an important component for the chemical analysis of cannabis and its many products. What follows is specific discussion on possible preferred analytical techniques for each of the chemical classes currently being measured. The intent is not to dissuade the use of other techniques, but to remind readers of the relative risks and benefits when certain alternative analytical techniques are employed.

## Potency: Cannabinoids

**Figure 1:**
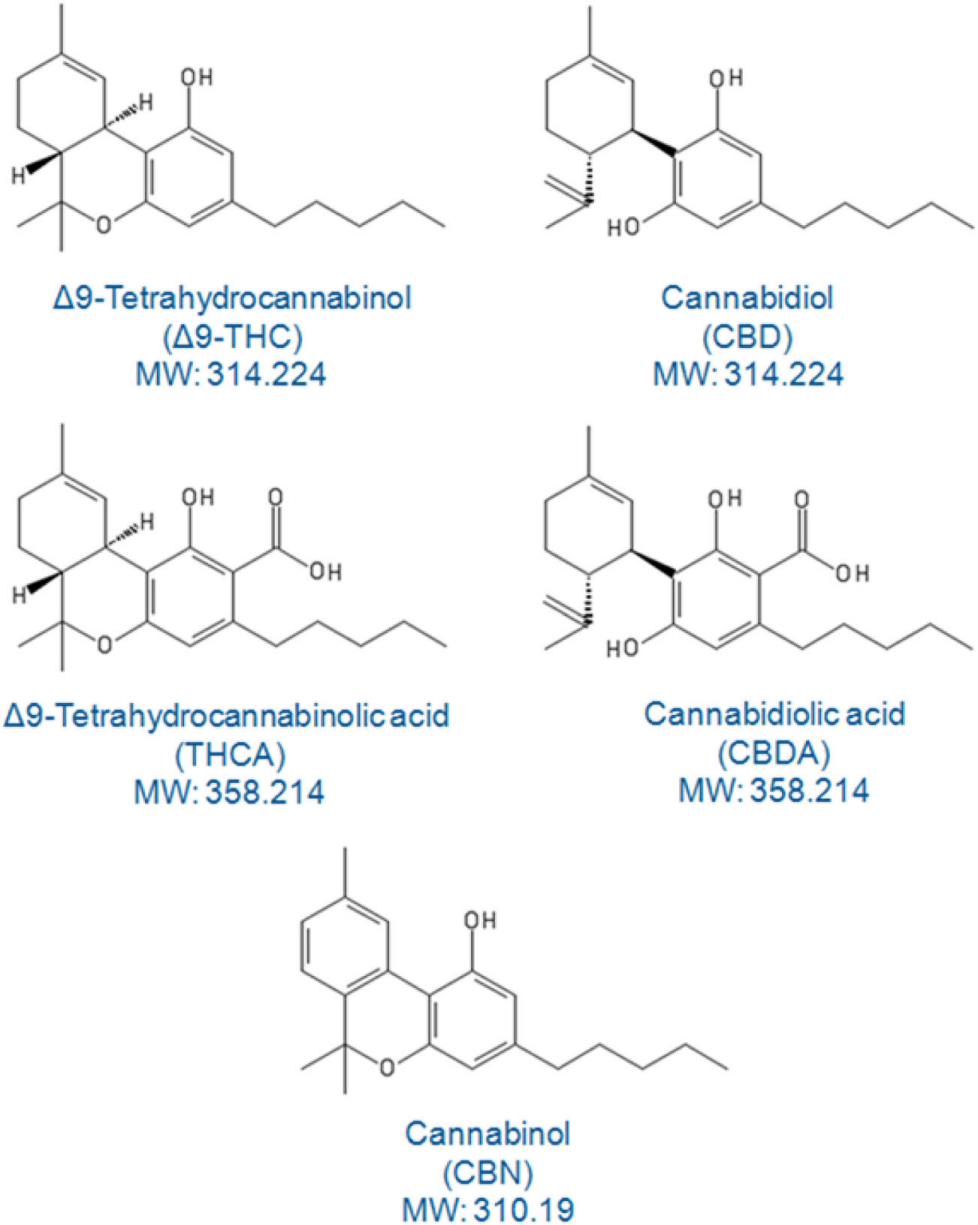
Structures of five cannabinoids commonly studied for potency

One of the most frequent measurements made for cannabis and hemp plant materials is “potency.” The potency term generally refers to the percentage of THC and or CBD (cannabidiol) in the plant material but in addition at least three other cannabinoids routinely monitored including THCA (delta-9-tetrahydrocannbinolic) acid, CBDA (cannabidiolic acid), and CBN (cannabinol). Figure 1 shows the structures of these related cannabinoid compounds. There are remarkable similarities between them, some being isobaric yet with dramatic differences in their pharmacological properties. More recently a total of eleven (11) different cannabinoids have been measured in cannabis plant materials [14] and the list may grow as new developments occur.

With consideration to the commercial “enthusiasm” for new cannabis products, it is important to recognize that the various cannabis strains or “cultivars” (the preferred term) contain a wide variety of other chemical constituents which are present in different ratios relative to each other as a function of the cultivar [15]. Thus, care must be taken with assuming the properties between different cultivars are the same since the ratios of many constituents differ between similar cultivars so chemical analysis must be performed to know the composition which can assure the expected medicinal effects.

There is growing evidence that the ratios of CBD relative to other cannabinoids as well as endogenous chemicals such as terpenes and flavonoids exhibit a desirable “entourage” effect [16] for medicinal benefits. Thus, it is the synergy between these other chemicals with the cannabinoids which may be responsible for the desirable medicinal outcome. There are at least 554 identified compounds in *C. sativa* L. plants which include 113 identified phytocannabinoids and 120 terpenesi [17]. Although it is believed that many of the cannabis plant constituents are important for a variety of reasons, the first question is the relative quantities of the major cannabinoids present. In contrast to modern pharmaceutical LC/MS bioanalytical trace analysis techniques, some of the cannabinoids are present in the plant at relatively high percent levels. It is common to dilute cannabis or hemp plant extracts by 100–1000-fold or more to reduce the concentration of targeted cannabinoids to levels appropriate for analytical quantitative determination. Analytical procedures need to be amended to maximize the information content obtained.

An important question for a cannabis grower is the “potency” of the plant. The composition of certain chemical constituents of the plant may have a large impact upon the value and expected price paid for that product by the buyer. A key component of accurate and precise analyses is specificity or “selectivity” of the detector used. In terms of analytical method validation, selectivity is defined as a method’s ability to measure and differentiate targeted analytes in the presence of other components that may be expected to be present [14]. The current popular analytical technique used in support of the cannabis industry for determining potency is HPLC/PDA, or HPLC using a photodiode array detector. This technology is accepted by the cannabis analytical community because it is relatively easy and inexpensive to employ. Although some may suggest that a PDA detector is relatively selective as an absorbance detector, there is growing evidence that it may not reveal potential co-eluting or potentially interfering endogenous plant chemicals since it does not provide the combined sensitivity and selectivity of a mass spectrometer detector [18]. An experienced plant biologist knows that a crude extract of a plant substance will contain a large number of chemical constituents. The HPLC/PDA analysis of this extract will likely include co-eluting chemical constituent(s) under the HPLC/PDA chromatographic peak(s). If this should occur, the area under that peak will be larger than it would be if only the targeted compound was contained in that peak. This increased peak area will produce a reported quantity of the cannabinoid that is higher than actually present and thus produce an incorrect result.

**Figure 2:**
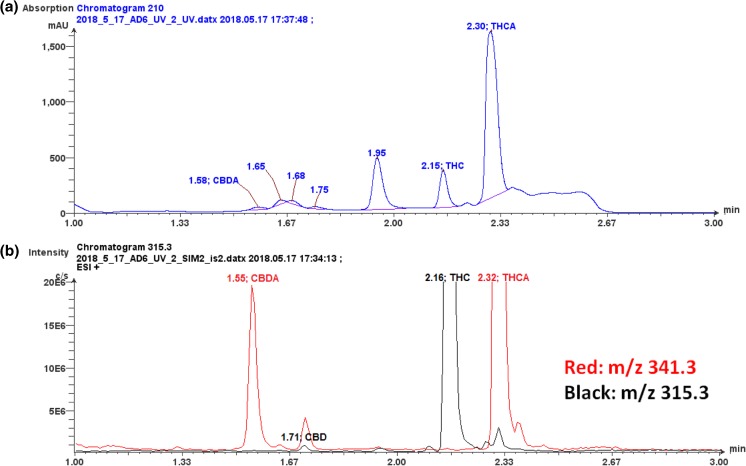
(**a**) LC/UV chromatogram of a cannabis extract showing expected abundant peaks for THC and THCA but other unknown chromatographic components which can interfere with the assessment of the quantity of other lower level cannabinoids. The multiple low-level peaks in the 1.4–1.9 min retention time region make it difficult to selectively quantitate cannabinoids which elute in this region. (**b**) LC SIM/MS analysis of the same extract shown in (**a**) where the protonated molecules for the targeted cannabinoids, CBDA, and CBD along with THC and THCA were monitored. Note: The *Y*-axis of (**b**) has been amplified by × 10 in order to clearly show the presence of the lower levels observed for CBDA and CBD. This naturally amplifies the THC and THCA peaks off scale as observed in (**b**) but clearly shows the presence of CBDA and CBD which were obscured in (**a**). The separation was achieved using an Agilent Eclipse Plus RRHD C18 (50 × 2.1 mm, 1.8 μm), mobile phase consisted of 0.1% formic acid in water and 0.1% formic acid in acetonitrile. The LC/UV chromatogram (**A**) was obtained at a wavelength of 210 nm and the LC/MS chromatogram (**b**) was collected using a single quadrupole mass spectrometer (Advion, Inc., Ithaca, NY) equipped with electrospray ionization under positive selected ion monitoring conditions

Figure 2 A, B shows a comparison analysis of HPLC/UV (Figure 2A) and HPLC/MS (Figure 2B) for a cannabis extract where it can be seen that the HPLC/UV chromatogram contains interfering peaks in the 1.5–1.7 min as well as the 2.4 to 2.6 min retention time region where some of the cannabinoids of interest elute. A low concentration of CBD is buried by the UV signals at 1.68 and 1.75 min retention times (Figure 2A). An inexperienced technologist could mistake the interference from the retention time peak at 1.68 or 1.75 min as CBD using HPLC/UV technology.

When selected ion monitoring (SIM) LC/MS analysis is performed on the same sample, the chromatogram shown in the lower frame of Figure 2B is obtained. SIM LC/MS analysis of this sample shows much improved selectivity and the absence of interfering chromatographic components. This is due to the increased selectivity of SIM LC/MS techniques where only the protonated molecules of the targeted cannabinoid(s) are monitored. Even higher selectivity coupled with mixture analysis capability could be obtained if LC SRM (selected reaction monitoring) MS/MS techniques, high-resolution time of flight, or Orbitrap mass spectrometry techniques were employed. The use of SIM LC/MS techniques shown in this example can reduce the potential for unknown interferences due to co-elution which may go unnoticed when HPLC/PDA techniques are used. Although it may be suggested that the use of ultra-high-performance liquid chromatography (UHPLC) 1.7-μm columns could provide higher separation efficiency, the limited selectivity of the PDA detector would still pose the potential for not differentiating between targeted and untargeted compounds. In addition, some of these chemical interferences are not yet fully characterized and lack commercial availability of certified reference standards. In contrast to HPLC/PDA, if either LC/MS or LC/MS/MS analysis of the same sample is performed, the considerably higher selectivity of either of these techniques could facilitate qualitative identification of unknown compounds as well as mitigate the potential for unexpected chemical interferences for the quantitative determination of the targeted compound [19].

The astute analytical chemist might suggest that prior HPLC/PDA analysis of a negative control plant sample could show where possible co-eluting plant chemical constituents may be observed such as those described in Figure 2A. The challenge here is the lack of an available negative control plant tissue since any cannabis or hemp sample will contain many of the targeted compounds of interest. In fact, most current methods used for potency testing by HPLC/PDA use a calibration curve with standards and quality control (QC) samples prepared in water or alcohol solvents which lack any plant matrix components. This approach to generating a calibration curve for the purpose of quantitative analysis is contrary to modern accepted bioanalytical techniques for the pharmaceutical or environmental industries and exposes the data to a variety of potential errors [20, 21].

## Pesticides

As cannabis is legalized for medical and recreational use on a state-by-state basis, safety regulations relating to cannabis products are becoming increasingly important. Pesticides rank high on the list of safety concerns in cannabis plants and the products derived from them. The level of pesticides and herbicides of course should be very low or absent since these could be present and/or concentrated in subsequent products as a result of the methods employed to produce products. Cannabis samples from shops and dispensaries in various states that have legalized the sale of recreational marijuana invariably reveal products contaminated with insecticides, fungicides, rodenticides, and other compounds used to eliminate or prevent insect or bacterial infestations [22]. Even though some cannabis is grown indoors within high-technology greenhouses with careful control of light and temperature, a variety of insect and bacterial pests are often found which can adversely affect the growth and quality of the plant before harvest. As a result, growers are tempted to use pesticides which are not allowed in their jurisdictions to control these problems. A good example of the breadth of pesticides of interest and their reporting requirements can be found in a recent Canadian guidance [23].

The US EPA has long been faced with setting and enforcing legal safe limits on toxic chemicals. Cannabis product safety is particularly relevant since medicinal cannabis can be used for young and immunocompromised patients as the intended consumers. Pesticide residues have evolved into a significant problem for the cannabis industry. A number of studies have reported high levels of pesticides on cannabis samples taken from medical markets in Colorado and Washington [22]. Recalls of cannabis products in both of these states and Canada (which recently legalized cannabis use nationwide) highlight the need for regulatory control. All this begs for routine chemical analysis of cannabis plants and products derived from them. An example of the toxic effects possible from pesticides comes from myclobutanil, a persistent fungicide used by some growers. When cannabis plant material containing myclobutanil [24] is smoked via a cigarette, it releases the very toxic gas, hydrogen cyanide. It is therefore important to provide reliable chemical analysis of plant material before it is consumed to ensure the absence of such pesticides. Each state has its own list of those pesticides which must be monitored in cannabis samples. California, for example, currently has a list of total 66 pesticides which must be monitored where 21 in this list are in category I and should not be detected in cannabis products while the other 45 pesticides in category II should not exceed indicated action levels at low part per billion (ppb) lower limits of quantitation (LLOQ) [25].

**Figure 3:**
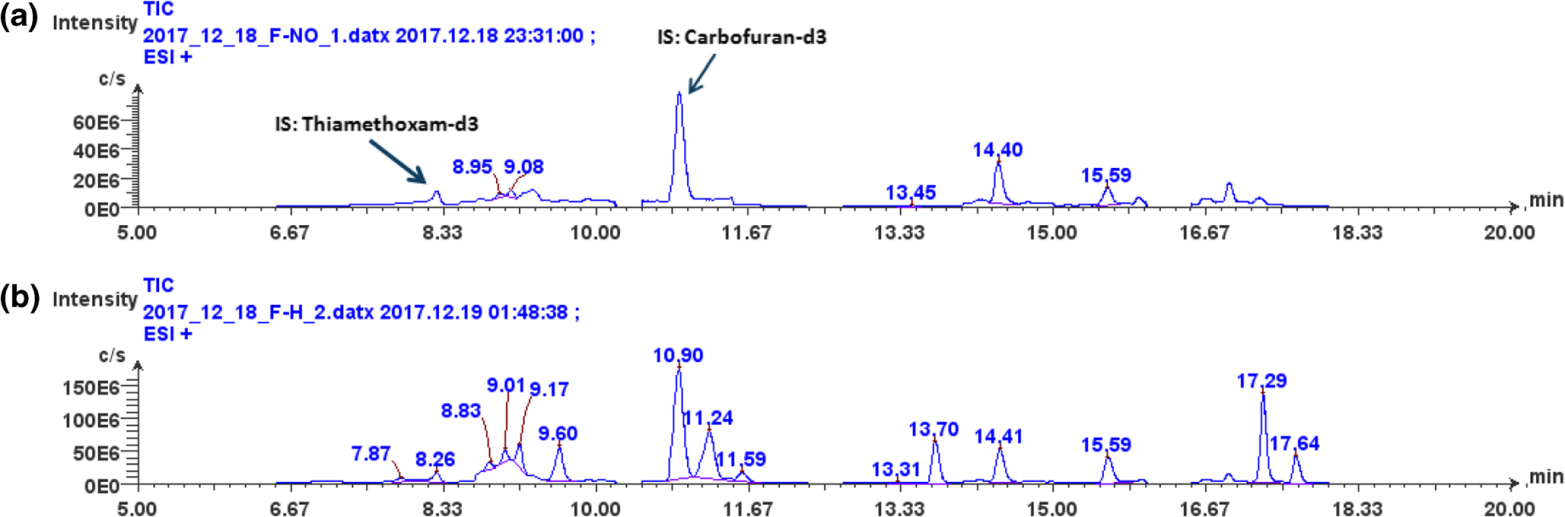
(**a**) SIM LC/MS of a marijuana plant sample which was believed to have not been exposed to pesticides. The plant extract was fortified with d3-thiamethoxam and carbofuran, respectively, which are observed at retention times of 8.26 and 10.90 min in (**a**). (**b**) The same sample in (**a**) which was sprayed in the laboratory with 15 known pesticides where the spray solution concentration was 100 μg/mL. The labeled peaks represent the 15 pesticides resulting from the sprayed marijuana plant. It can be noted that some of these pesticides were in fact present in the extract shown in (**a**) SIM LC/MS chromatogram. These included imidacloprid (RT = 8.95 min); acetamiprid (RT = 9.08 min); chlorantraniliprole (RT = 13.45 min); Systhane (RT = 14.40 min); and propiconazole (RT = 15.59 min). Table 2 lists the 15 pesticides and their observed retention times in (**a**, **b**). Table 1 lists the 15 pesticides and their observed retention times in this figure. The pesticide separation was achieved using an Agilent Eclipse Plus RRHD C18 (50 × 2.1 mm, 1.8 μm) with a mobile phase consisting of 0.1% formic acid in water and 0.1% formic acid in methanol. The chromatograms were obtained using a single quadrupole MS (Advion, Inc., Ithaca, NY) coupled with electrospray ionization under positive selected ion monitoring conditions. The authors acknowledge Dr. George Maylin of the New York State Drug Testing Program for providing the cannabis plant sample and accommodating our acquisition of these data in his licensed controlled substance laboratory

The chemical complexity of cannabis plant materials and products produced from them coupled with the regulations for low ppb levels can benefit from the analytical capabilities of modern separation science coupled with mass spectrometry. Although GC/FID analyses have been employed in the past for measuring some pesticides, more recently, GC/MS, GC/MS/MS, LC/MS, or LC/MS/MS techniques may be preferred. Figure 3A, B shows an example of SIM LC/MS analysis of a plant extract containing pesticides using electrospray ionization (ESI) as an approach for the determination of pesticides in cannabis plant matrix extracts. In this example, a flower portion of a cannabis plant was extracted and the extract fortified with two stable isotope incorporated internal standards (d3-thiamethoxam and d3-carbofuran) followed by SIM LC/MS analysis while monitoring the protonated molecules of 15 common pesticides (Table 1). Due to the relatively high selectivity of this SIM LC/MS analytical approach, the sample can be screened for targeted pesticides in the sample. As an example, Figure 3B shows SIM LC/MS results for a cannabis flower sample which had been sprayed with a solution containing 100 μg/mL of 15 pesticides followed by extraction in the same manner as the control sample shown in Figure 3A. Each of the 15 pesticides sprayed onto the cannabis flower (see Table 1) is detected in the cannabis extract as denoted by their labeled retention times in Figure 3B.

**Table 1:** The Fifteen Pesticides Observed in Figure 2B Listed in Order of Their Retention Times

Pesticides	RT (min)
Methomyl	7.87
Thiamethoxam	8.26
Imidacloprid	8.83
Dimethoate	9.01
Acetamiprid	9.17
Thiacloprid	9.60
Carbofuran	10.90
Ancymidol	11.24
Carbaryl	11.59
Chlorantraniliprole	13.31
Azoxystrobin	13.70
Systhane	14.41
Propiconazole	15.59
Etoxazole	17.29
Fenpyroximate	17.64

It is interesting to note that five of the pesticides sprayed onto the cannabis flower observed in Figure 3B were already present in the sample shown in Figure 3A prior to the pesticide spray process (see Figure 3A, B legend for the name and retention times observed in Figure 3A of these five pesticides). This is not surprising in the wake of a report by an accomplished laboratory which reported that 83% of samples they analyzed in 2017 contained one or more pesticides [26]. With increased testing of cannabis-derived samples for pesticides, numerous detections of low-to-moderate levels of pesticide contamination (10’s of ppb or less) from a number of growers claiming to use “organic” or “clean green” growing methods prompted the investigation of possible sources. The most prevalent pesticide detected in these cases was myclobutanil, most commonly used as a treatment for mold infestations [26]. In many cases where pesticide levels are in the mid-to low ppb range, this SIM LC/MS approach may be appropriate. For those samples containing very low ppb levels of unknown pesticides, it may be preferred to employ LC/MS/MS techniques or high-resolution MS employing a QTOF or Orbitrap. A recent report compared the range of pesticides amenable to GC/MS vs. those amenable to LC/MS/MS and it was concluded that GC/MS only covered a few of more than 500 pesticides studied due to their thermal instability or involatility [27]. Electrospray LC/MS/MS techniques covered the majority of those pesticides studied, although some analyses may still benefit from modern GC/MS techniques to compliment the LC/MS/MS approach. In general, most state laboratories, including New York state, suggest the use of LC/MS/MS techniques for the quantitative determination of pesticides in cannabis, hemp, and the products derived from them [28]. This technology coupled with recommended sample preparation provides broad coverage with high sensitivity and selectivity for the quantitative determination of over 100 pesticides.

## Terpenes

Throughout the plant kingdom, the biosynthesis by botanical epidermal features called trichomes (vide infra) produce cannabinoids [2] but also a wide range of terpenes containing just carbon and hydrogen (these are the predominant terpenes in cannabis) as well as oxygenated neutral compounds including terpene alcohols and ketones as well as some aldehydes and esters [2]. Most of these compounds have a relatively high vapor pressure and thus give off a pleasant odor from the plant. The familiar smell of a fresh cannabis or hemp plant is due in part to these terpene-related compounds [2]. Terpenoids are not unique to cannabis, but various types of cannabis plants produce unique terpene profiles which in combination with the unique ratios of cannabinoids may determine some of the preferred medicinal effects [8]. It is the determination of these chemical profiles that requires chemical analysis which has generally been done by capillary GC/FID or more recently capillary GC/MS for this complex mixture of over 200 different terpene compounds. The very high separation efficiency of capillary GC columns coupled with the generally universal ionization efficiency of electron ionization (EI) mass spectrometry would appear to be a preferred analytical technique for the determination of these compounds. In contrast, however, there is a recent report employing LC/MS/MS techniques with atmospheric pressure chemical ionization (APCI) [24]. This approach may not have the optimal chromatographic separation efficiency compared to capillary GC columns, but when combined with the mixture analysis capabilities of tandem mass spectrometry may produce the results needed while also being amenable to some of the other important cannabis compounds in the same sample.

Since terpenes are not cannabinoids, some feel these are not important constituents of a cannabis plant or product. However, there is increasing evidence that other chemicals in the plant work synergistically with the cannabinoids that produce therapeutic responses ranging from lowering of inflammation and regulation of blood glucose to protection of neurons and gastric cells [8]. As mentioned above, this is the so-called entourage effect [16] where other chemicals may enhance or moderate the effects of the THC, CBD, etc. For example, one report suggests that CBD may be an “entourage” compound in cannabis that modulates the effects of THC [16]. Therefore, the quantitative determination of terpenes and sesquiterpenes is often desired and perhaps best done by either GC/MS or LC/MS/MS instead of some of the earlier reported techniques [2].

## Heavy Metals

Phytoremediation refers to procedures which employ plants to scavenge heavy metals from the environment [29]. For agricultural crops, this procedure is sometimes used prior to seeding the farm land for food products. This technique depends upon the ability of certain plants to sequester heavy metals from the soil so that when food-producing crops are planted, there will ideally be no heavy metals in that crop [30]. As it turns out, cannabis plants are remarkably capable of removing heavy metals from the soil without affecting their own heartiness [31]. This of course concentrates these heavy metals in the cannabis plant materials where they can later reside in products derived from the cannabis plant. While some metals are beneficial and essential for life, others are highly toxic and have negative effects on good health. Whether the dry cannabis plant’s leaves are smoked or medicinal products are produced, the user may be exposed to unhealthy levels of potentially toxic heavy metals. As a result, it has become standard practice to analyze cannabis plant materials as well as products derived from them for a number of these heavy metals. Among those heavy metals, most commonly determined are lead, cadmium, mercury, and arsenic although depending upon which states are involved this list may be longer. A number of studies suggest that cannabis is an active accumulator of additional metals including magnesium, copper, chromium, and cobalt. These metals are often found if the plant is grown near a mining, smelting, or sewage sludge sources [31].

Cannabis testing laboratories are adding sample preparation and analysis procedures to their services which can quantify a growing list of heavy metals found in plant and the derived products. Cannabis can be tested for heavy metals in many ways, such as various forms of atomic spectrometry, including atomic absorption(AA), inductively coupled plasma optical emission spectroscopy (ICP-OES), and inductively coupled plasma mass spectrometry (ICP-MS) [32]. For AA, the method used would have to be the more sensitive graphite furnace atomic absorption (GFAA) since the flame AA method would not be sensitive enough for most elements. Also, mercury must be measured by a method called cold vapor atomic absorption spectroscopy (CVAAS) due to the lower sensitivity by AA [32].

In general, flame techniques can measure elements at low parts per million, and GFAA goes down to low parts per billion. Also, the AA method usually measures one element at a time. ICP/OES and ICP/MS are techniques capable of measuring multiple elements simultaneously and are thus preferred where multi-heavy metal measurements are desired. Nonetheless, using ICP/OES to test cannabis for heavy metals often requires a way to enhance its sensitivity, such as introducing the sample with an ultrasonic nebulizer (USN). Reports have shown that USN can increase sensitivity up to a factor of 10 in many cases [33]. Experienced analytical chemists generally report that ICP/MS offers the best sensitivity and is the method used in many modern laboratories. For some important organometallic compounds, it can be useful to employ LC/ICP/MS techniques which can allow detection and quantitation of the individual organometallic compounds. The Food and Drug Administration (FDA) and United States Pharmacopeia (USP) have standardized methods for heavy metal analysis, which are very useful resources in the fledgling cannabis-testing industry, where regulation has been a bit slow to catch up [33].

## Microbiology

Cannabis is often grown in greenhouses with carefully controlled growing conditions which are warm with relatively high humidity; these are excellent conditions for the growth of a wide variety of bacteria and fungi. As a result, the final cannabis product may be contaminated with these organisms, which can adversely affect the safety of the eventual products produced from these plants. Accordingly, most states now require testing laboratories to analyze samples for microbial growth. Microbiological contamination of cannabis plants and products is typically detected using culture growth or quantitative polymerase chain reaction (qPCR) techniques. Currently, mass spectrometry is not generally used for identifying bacteria and fungi in complex samples, although MALDI MS is well-suited for the qualitative identification of these organisms in pure culture or simple matrices. Further studies could perhaps demonstrate the potential for MALDI identification of bacteria and fungi in cannabis samples, as is currently used in the clinical laboratory environment [34].

## Mycotoxins

Mycotoxins are toxic secondary metabolites of mold which result from long-term storage of organic plant material. They are a concern in cannabis plants because of the suspected carcinogens causing acute and chronic toxicity [8]. Aflatoxins are a subset of mycotoxins produced by *Aspergillus flavus* and *Aspergillus parasiticus*. Aflatoxin B1 is considered the most toxic, but the presence of B2, G1, and G2 must also be considered as they result from decaying vegetation and soils when warm, moist conditions exist. There are regulations now that set limits on the allowable levels of these toxins in foods. Both recreational and medicinal cannabis must now be screened for mycotoxins derived from microbial contamination. This can be done by a number of analytical techniques, but due to the chemical complexity of the sample extracts and the polar and/or labile nature of some mycotoxins, they are good candidates for LC/MS and LC/MS/MS analysis [35].

## Residual Solvents

Due to a variety of extraction processes used for isolating cannabinoids from the cannabis plant, residual solvents often remain in the sample. Since many of the targeted cannabinoids are very non-polar, the preferred extraction media are non-polar compressed gasses and solvents. These solvents include hexane, ethanol, butane, propane, and in some cases chlorinated solvents as well as supercritical carbon dioxide [8]. Many states require testing for these residual solvents which is often done by headspace GC/FID. The sample preparation for these samples is relatively simple where a small amount of the extract is placed into a vial and heated to mimic the natural evaporation process. The amount of solvent that is evaporated from the sample and into the air represents the “headspace.” An aliquot of this is analyzed by GC/FID. Although an alternative technique could be EI GC/MS analysis of such headspace samples, the reduced equipment costs of GC/FID may likely prevail for the foreseeable future.

## Determination of Origin of Growth of Cannabis Using Isotope Ratio Mass Spectrometry

Federal and local agencies are often interested in a drug’s origin and in determining large-scale trafficking routes for potential legal/enforcement solutions. *C. sativa* L. can reveal its photosynthesis activities by recording the isotope ratio of carbon [36]. Environmental factors such as climate, water availability, temperature, and light intensity have an impact on the assimilation of carbon 13 in the plant and its contents. Plant tissues are depleted with respect to the atmospheric source because photosynthesis and enzymatic fixation are discriminating against the heavier isotope. The photosynthetic pathway for cannabis is known depending upon the growth conditions. The ratio of CO_2_ uptake in the plant to the concentration in the air is controlled as part of the photosynthetic activity. Isotope ratio mass spectrometry (IRMS) has been used to accurately determine the carbon 13 content of cannabis plants and can be used as a forensic tool to establish the geographic origin of the plant’s growth. Although this analytical technology is not commonly employed in routine analytical services for the cannabis industry, it has certainly proven itself in those instances where it is important to know the cannabis plant’s geographic origin if needed [37]. This reference describes how the stable carbon and nitrogen isotopic ratios were measured in marijuana samples (*Cannabis sativa* L.) seized by the law enforcement officers in three Brazilian production sites: It was possible to differentiate samples from the dry regions from those from regions that are regarded as different with respect to heavier rainfall. The results were in agreement with the climatic conditions of the suspected regions of origin and this demonstrated that seized samples can be used to identify the isotopic signatures of marijuana from the main producing regions in Brazil [37].

## Biological Sample Analysis: Forensic Toxicology

Currently, most of the state cannabis-testing laboratories are not performing analyses of biological samples for the quantitative determination of cannabinoids or their metabolites. These applications could be for clinical toxicology purposes to determine if cannabis contributed to observed toxicity, or for forensic toxicology purposes, to determine unlawful use of performance-altering substances. The topic of driving under the influence of drugs (DUID) raises increasing concerns by authorities such as the National Safety Council’s Alcohol, Drugs and Impairment Division (NSC-ADID) [38] and the National Highway Transportation Safety Administration (NHTSA) [39] as well as the general public. Recent studies on driving impairment due to cannabis use and/or combined with alcohol consumption showed increased risks of driver-induced automobile crashes [40].

Blood THC and metabolite concentrations are greatly hampered by the delay in time required to collect the samples after a police stop or crash. Historically, sample analysis was performed by GC/MS which required chemical derivatization and relatively long run times. Since urine analysis for delta-9-THC and its metabolite, carboxy-THC, have not generally shown an association between cannabis and automobile crash risk [41], blood or plasma analyses as well as oral fluid analyses [40, 42, 43] have been studied for DUID applications. A number of challenges exist with both of these approaches. The non-psychoactive cannabinoid, THC-COOH, can be detected for several days depending upon the dose and mode of administration while psychoactive THC can last for many days from chronic smokers in the blood but there is little correlation between THC in blood and driving impairment [43]. With current delays due to the lack of facile on-site blood collection, the THC level in a smoker’s blood will be very low several hours after the incident making it difficult to relate “under the influence” with the biological fluid levels at the time of analysis.

**Figure 4:**
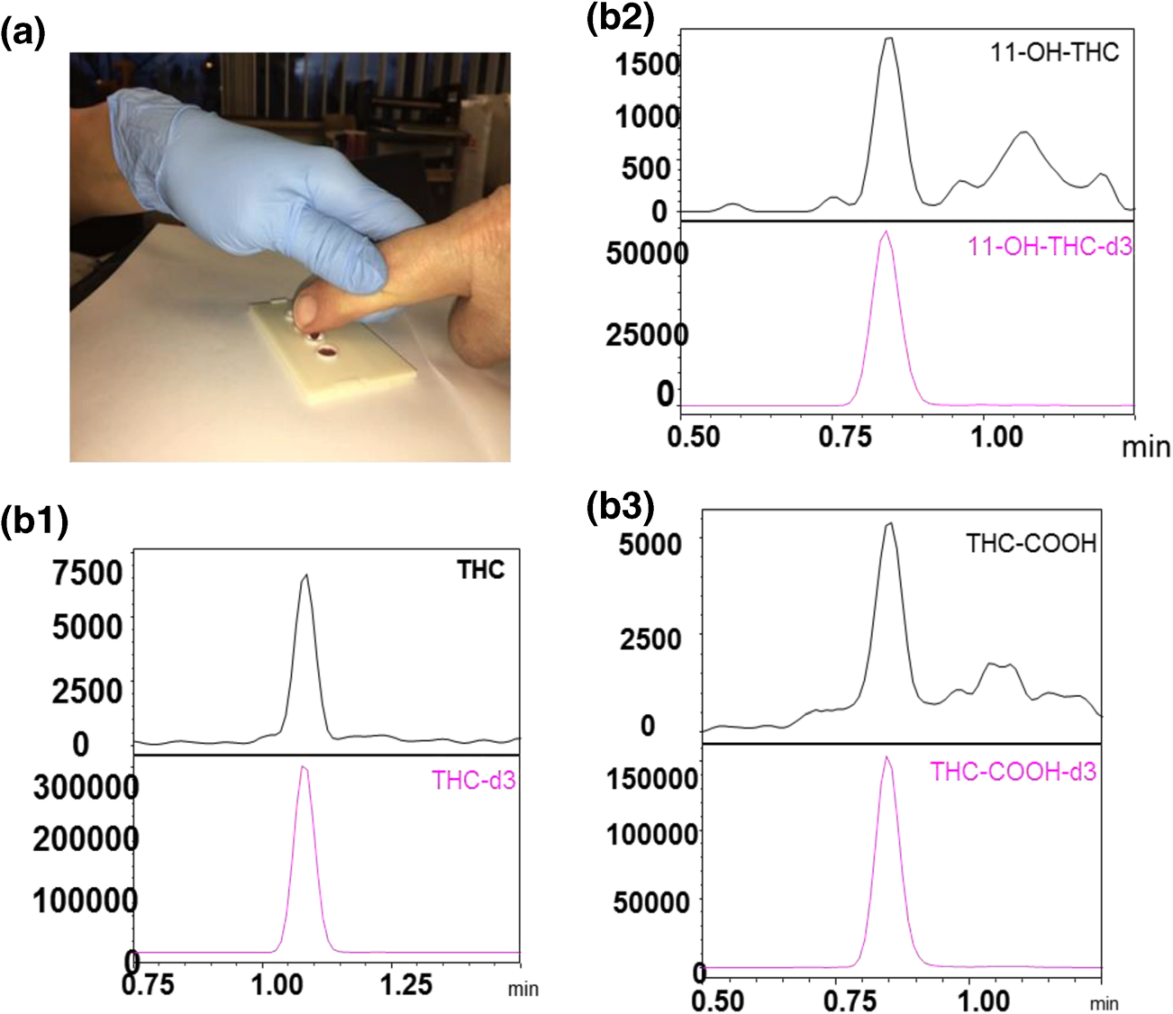
(a) Finger prick collection of blood to produce a dried plasma spot. (b1–b3) SRM LC/MS analysis of dried plasma spot from a marijuana smoker showing the presence of the parent compound, THC, along with its two main metabolites, THC-OH and THC-COOH. The lower magenta traces in each panel are the SRM LC/MS chromatogram for the corresponding deuterated internal standard for each compound. Experimental details for this approach are described elsewhere [46]. The labels below of b and bc are incorrect; they should be b1 and b2. Also, they are lower case in the figure but referenced in the text in upper case. Shouldn't they be consistent?

In an effort to provide a potential solution to these issues, we and others have considered the potential for micro-sampling with dried blood spots (DBS) [44, 45] and dried plasma spots (DPS) [46]. In other studies, the potential for oral fluids [42] as well as breath analysis [47] continue to be evaluated as a means for facile sample collection coupled with efficient transport to a forensic laboratory. An example of on-site finger prick blood onto a dried plasma spot card is shown in Figure 4(A) where a single drop of blood from regular cannabis users was collected on-site at the 2017 Cannabis Science Conference (see Figure 4(A)). The book-type DPS [46] card filters the red blood cells from a drop of blood to produce a separate small sample of red blood cells along with a separate dried plasma spot from the same sample. This approach is a fast, on-site sampling technique that minimizes analysis delays and stabilizes THC and its metabolites because the samples are dry. After a 20-min drying period with the two “spots” separated from each other by opening the device 180° via the book hinge’, the device was sent via courier cross-country to the laboratory where the DPS samples were analyzed by SRM LC/MS.

Figure 4(B1–B3) shows the SRM LC/MS ion current chromatograms for delta-9-THC and its two metabolites, carboxy-THC (THC-COOH) and hydroxy THC (11-OH-THC), determined from the finger prick of a person who had recently smoked a marijuana cigarette. The lower magenta traces in each panel of Figure 4(B1–B3) are the SRM LC/MS chromatograms for the corresponding deuterated internal standards for each compound which provided high-quality quantitative determination of these three compounds. Table 2 shows the quantitative results for those cannabinoids detected in samples collected from a group of different cannabis users that did not reveal their mode of administration. However, sample 5 shown in Figure 4(B1–B3) as noted above was provided by a person who had recently smoked marijuana followed by a finger prick collection of the blood to provide a dried plasma spot. From Table 2, it can be seen that only this person (No. 5) had detectable levels of the two main metabolites of THC, namely THC-COOH and 11-OH-THC. It is likely that future adaptations for sample collection of biological fluids will evolve with the preferred chemical bioanalysis technique being LC/MS/MS or LC-high-resolution MS.

**Table 2:** SRM LC/MS Analysis of Dried Plasma Spots Collected as Finger Pricks from “Users” of Various Cannabis Products. Sample #5 Is the Only One Who Had Recently Smoked Marijuana Prior to Blood Collection, and Thus, the Sample Contained the Predominant Metabolites of THC Which Include THC-COOH and THC-OH

Sample no.	THC (ng/mL)	THC-COOH (ng/mL)	THC-OH (ng/mL)
1	157.7	ND	ND
2	189.5	ND	ND
3	53.9	ND	ND
4	46.6	ND	ND
5	527.6	180.8	97.3
6	284.7	ND	ND

*ND* not determined

## Challenging Sample Preparation Issues: Ambient Ionization Mass Spectrometry

It should be evident from above that there is a wide diversity of sample types, sample matrices, and targeted compounds for which chemical analysis is needed. These matrices range from marijuana and hemp plant materials, biological samples including blood, urine, oral fluids, and breath condensate to commercial products such as medicinal oils and salves. In addition, edible foods ranging from brownies to gummy bears as well as wine and other drinks are appearing on the commercial market. It is obvious to an experienced analytical chemist that the approach to sample preparation will vary as a function of the sample matrix, the targeted chemicals, and the necessary detection limits. The latter is particularly important since the majority of the chemical analyses require quantitative analysis which in the case of pesticides require lower limits of quantitation (LLOQ) of a few tens of parts per trillion in some cases. Thus, sample preparation will not likely be a one approach that fits all matrices for cannabis chemical analysis. Recent developments in QUECHERS offer some promise as well as some shortcomings [48]. It should be apparent there is opportunity to develop novel sample preparation techniques that can simplify and/or improve our strategy for the determination of the above-described compound classes in cannabis plant materials and products.

It is not uncommon in a new market for some who will want very simple approaches to chemical analysis, often suggesting a “method” that does not involve any sample preparation at all. This is the Holy Grail for many who do not wish to perform multi-step sample preparation or HPLC separations. In support of this desire, there has been on-going interest in direct analysis of untreated samples using mass spectrometry. In recent years, several ambient ionization techniques have been reported along with some selected successful applications [49]. These techniques include DART (direct analysis in real time), DESI (desorption electrospray ionization), and ASAP (atmospheric solids analysis probe). In principle, these techniques provide a means, for example, of touching a glass capillary probe (for DART and ASAP) onto the surface of an untreated sample followed by direct introduction of that probe into the API (atmospheric pressure ionization) region of a mass spectrometer. The challenge associated with this approach is the lack of component separation provided by chromatography in LC/MS applications and the frequent need for quantitative determination of both major and minor components. As a result, the noted ambient ionization techniques have not been competitive to date with LC/MS, LC/MS/MS, GC/MS, GC/MS/MS, or high-resolution mass spectrometry techniques for the quantitative bioanalytical determination of components in complex biological matrices. However, it may be that these techniques can play a role in preliminary screening techniques for targeted compounds in hemp and marijuana samples going forward.

**Figure 5:**
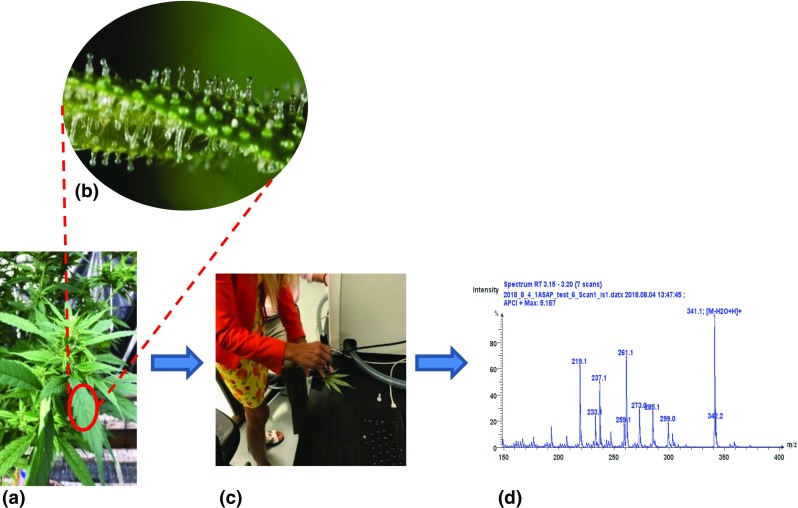
(**a**) Photo of a typical cannabis plant. (**b**) Magnification of a cannabis leaf surface showing the trichomes on the surface of the leaf (https://honestmarijuana.com/trichomes/). (**c**) Analyst “swabbing” the surface of a cannabis leaf with an ASAP probe for ASAP MS using positive ion APCI. (**d**) ASAP MS full-scan APCI mass spectrum from a swab of a cannabis leaf with the ASAP glass capillary probe which has been subjected to thermal desorption from the glass surface by heated nitrogen gas. Experimental conditions: single quadrupole compact mass spectrometer (Advion, Inc., Ithaca, NY), positive ion APCI source with an inlet capillary temperature of 250 °C, capillary voltage 150 V, source offset 40 V to promote in-source collision-induced dissociation, nitrogen gas temperature 350 °C, and a corona discharge of 5 μA

An example relevant to the cannabis industry is shown in Figure 5(A–D). This includes a photograph of a hemp plant (Figure 5(A)) followed by another which shows the microscopic resin glands on the plant which are called “trichomes” (Figure 5(B)) on the leaves of a cannabis plant. Derived from the Greek word *trikhōma*, which means “growth of hair,” trichomes are nearly microscopic, mushroom-like protrusions from the surface of the buds, fan leaves, and—in lower numbers—even on the stalk [50]. While relatively complex, trichomes are comprised primarily of a stalk and a head. It is within the head that the actual production of cannabinoids like THC occurs. These trichomes are the “chemical factories” of the cannabis plant where most of the seemingly more interesting chemical constituents of the plant are located which include the cannabinoids, terpenes, and others. Thus, for the analyst who wishes to obtain preliminary mass spectral information from the cannabis plant, a simple “swab” of the plant leaf surface with a DART or ASAP (Figure 5(C)) probe can provide an APCI (atmospheric pressure chemical ionization) “mixed mass spectrum” of what is in the trichomes (Figure 5(D)). This may preclude issues of interferences from chlorophyll or other complex chemicals found elsewhere in the cannabis plant. Similarly, if one wishes to employ DESI to obtain ESI mass spectra, a small portion of plant material containing the trichomes may be placed on the sample surface for DESI MS analysis.

## Summary

We are at a very early stage of the future cannabis industry. Movement towards acceptance of cannabis has been glacially slow due to regulatory issues, but there may be a tidal wave of the industry’s growth in the near future as recently evidenced by Canada legalizing cannabis nationwide. There is an urgent need for accurate and precise chemical analysis of large sample numbers ranging from cannabis plant materials to final products including medicinal products, foods, and biological fluids. Going forward, it will be important to assure users of the products that safe, accurate levels of expected active ingredients are present as well as to assure them that unsafe chemicals including pesticides, mycotoxins, and heavy metals are absent. Although some market participants would like a simple handheld device such as a Raman spectrometer [51], this or other technology has not yet demonstrated its potential successfully in the cannabis industry, but perhaps it will find a role in the future. The driver for this is the continued interest in an easy-to-use device for measuring the qualitative and quantitative composition of cannabis plants and products.

Although mass spectrometry techniques are currently used to some extent in the cannabis industry, we suggest it should be used more widely in the future to provide the superior combination of selectivity and sensitivity demanded by the chemical complexity and diversity of the plant and the many products likely to be produced from it. This will be necessary for the production and sale of safe products and to earn customer confidence and acceptance in those products. An important component of this is the use of modern analytical chemistry techniques and procedures which include standard operating procedures (SOPs) and rigorously validated methods. This will require well-trained analytical chemists who practice sound scientific procedures common to laboratories following good laboratory practices (GLP) and anti-doping laboratories that abide by well-recognized accredited laboratory practices [52]. Good examples to follow are those recommended by good laboratory procedures (GLP) as well as approved methods and guidance provided by well-established organizations such as the ISO/IEC 17025 [53], AOAC (Association of Official Agricultural Chemists) [54], A2LA (American Association of Laboratory Accreditation) [52], and individual state health departments [28]. When well-qualified analytical service laboratories are in place for monitoring the chemical safety and integrity of cannabis materials and products, the industry may thrive beyond our current level of appreciation.
